# Pulse Reshaping in Double-zero-index Photonic Crystals with Dirac-like-cone Dispersion

**DOI:** 10.1038/s41598-020-65461-8

**Published:** 2020-05-21

**Authors:** Tao Xu, Dejun Zhu, Zhi Hong Hang

**Affiliations:** 1 School of Physical Science and Technology & Collaborative Innovation Center of Suzhou Nano Science and Technology, Soochow University, Suzhou, 215006 People’s Republic of China; 2 Institute for Advanced Study, Soochow University, Suzhou, 215006 People’s Republic of China

**Keywords:** Photonic crystals, Metamaterials

## Abstract

Triply-degenerate Dirac-like cone at the Brillouin zone center attracts much research interest in recent years. Whether the linear dispersion in such a Dirac-like cone reflects the same physics to Dirac cones at the Brillouin zone boundaries is still under investigation. In this manuscript, through microwave experiments and numerical simulations, we observe intriguing pulse reshaping phenomena in double-zero-index photonic crystals, which cannot be fully understood from their close-to-zero effective parameters. A reshaped pulse, with frequency components close to the Dirac frequency filtered, is propagating at a constant group velocity while part of these filtered frequencies appears at a much later time. In time domain measurements, we find a way to separate the effect between the linear dispersion and the extra flat band in Dirac-like cone to have a better understanding of the underneath physics. We succeed in obtaining the group velocity inside a double-zero-index photonic crystal and good consistence can be found between experiments, numerical simulations and band diagram calculations.

## Introduction

In graphene, the conduction and valence bands touch each other at a singular point, Dirac point, and a conical band diagram, Dirac cone, is formed at the Brillouin zone (BZ) boundary. Dirac cones are described by two obbligato features: a degenerate Dirac point and the linear dispersions near the Dirac point. Not restricted to graphene, with a similar honeycomb lattice, Dirac cone dispersions at the BZ boundaries have been observed in various artificial periodic structures, such as photonic crystals (PCs)^1–4^, sonic crystals^4–6^, and metamaterials^7^. Near the Dirac point inside the PCs, the Maxwell equations can be reduced to the Dirac equation, and some unusual transmission properties such as pseudo-diffusion^1,8^ occurs. The transmission through the PC is inversely proportional to the thickness of the sample. In addition, with a pulse incidence, extraordinary pulse reshaping occurs in photonic and acoustic crystals with Dirac dispersions^9,10^ which is considered to be a classical analog of the Zitterbewegung phenomena, the trembling motion of wave packages. Moreover, by introducing a symmetry breaking to the Dirac cone at the BZ boundaries, valley-induced unidirectional electromagnetic propagations are realized^11–14^.

**Figure 1:**
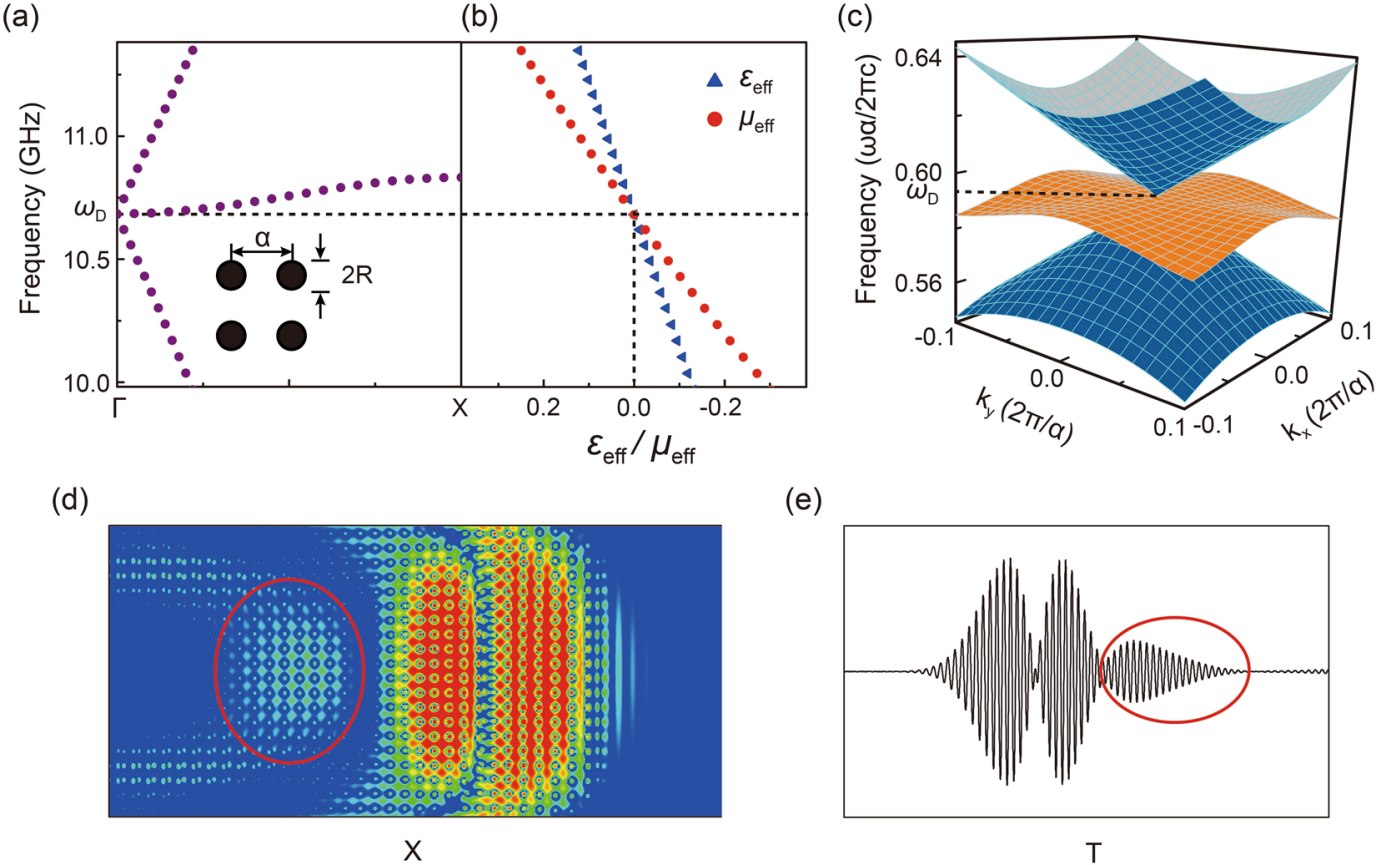
(**a,b**) TM photonic band diagram and effective permittivity/permeability shown in blue triangles/red circles near the Dirac frequency *ω*_*D*_ for a 2D square-latticed alumina cylinder array in air. (**c**) 3D TM band contour for the 2^nd^, 3^rd^ and 4^th^ bands of such a PC. (**d**) Simulated electric field amplitude distribution of a Gaussian pulse incident from the left. (**e**) The received temporal evolution of the finite pulse at a probe point outside the PC.

In 2011, Huang *et al*. proposed a double-zero-index (DZI) PC by using a square array of dielectric cylinders^15^. By precisely tuning the filling ratio of the PC, a triply degenerate Dirac-like-cone dispersion appears at the BZ center, as can be seen in Fig. 1(c). Accident degeneracy plays a key role to form a Dirac-like cone while it is the lattice symmetry guarantees the existence of Dirac cone at BZ boundaries^4^. The effective DZI parameters with both permittivity and permeability to be zero can be retrieved from such a PC at the corresponding Dirac frequency *ω*_*D*_. However, located at BZ boundaries, effective parameter retrieval to PC with Dirac cone is impossible. With the help of the wave tunneling property of DZI materials, cloaking in the waveguide^15–19^, wavefront engineering^20,21^, coherent perfect absorption with configurability^22^ and transmissive invisibility cloaking^23^ are proposed and demonstrated. As only pure dielectrics with reasonable permittivities are needed, the similar principles have been extended to higher frequencies^24,25^ and even acoustic regime^26^. By inducing a band inversion between the doubly-degenerate dipole mode and monopole mode to form the accidental degeneracy of the Dirac-like cone, deterministic interface states^27–29^ are achieved which can be related to the Zak phase of PCs^30–32^. There seems to have more difference between Dirac cone and Dirac-like cone. The berry phase of Dirac cone is calculated to be *π* while a 0 value is obtained for Dirac-like cone^4,5^. Moreover, how an electromagnetic pulse evolves when propagating through a system with a Dirac-like-cone dispersion has never been discussed.

In this manuscript, we consider a two-dimensional (2D) square lattice of cylinders immersed in air with a lattice constant *a* = 16.63 mm, with Transverse Magnetic (TM) polarization. The electric field is polarized along the z axis, parallel to all cylinders. The radius (R) and the dielectric constant (ε) of the cylinders are 0.2255*a* and 8.2, respectively. A Dirac-like cone with three bands degenerate at Dirac frequency ${\omega }_{D}=10.682\,{\rm{GHz}}$ can thus be achieved, as shown in Fig. 1(c). Different from the Dirac cone at the BZ boundaries, the Dirac-like cone at the BZ center is with an extra flat band intersecting the Dirac cone at the Dirac point. A part of the calculated photonic band diagrams and the corresponding effective permittivities and permeabilities close to *ω*_*D*_ of the PC are shown in Fig. 1(a,b) respectively. We impinge a Gaussian pulse with both spatial and spectral distributions into the PC from left in the simulation and the distribution of such a pulse inside our DZI PC is obtained. Though the phase of the electric field inside the PC is nearly homogenous, representing its close-to-zero property, what interests us is that the pulse is divided into three parts, as can be clearly seen in Fig. 1(d). We also record the temporal evolution of such a pulse at a point outside the DZI PC [as shown in Fig. 1(e)] and it represents the same pulse reshaping phenomenon: three different pulse packages arrive the probe at different time. When we replace the PC with its effective parameters, as illustrated in Fig. 1(b), we obtain a different phenomenon: the part enclosed by a red ellipse vanishes, as previously reported^33,34^. We thus conduct a detailed analysis, in both experiments and simulations, on such a pulse reshaping phenomenon.

## Results

### Pulse reshaping in DZI PCs

**Figure 2:**
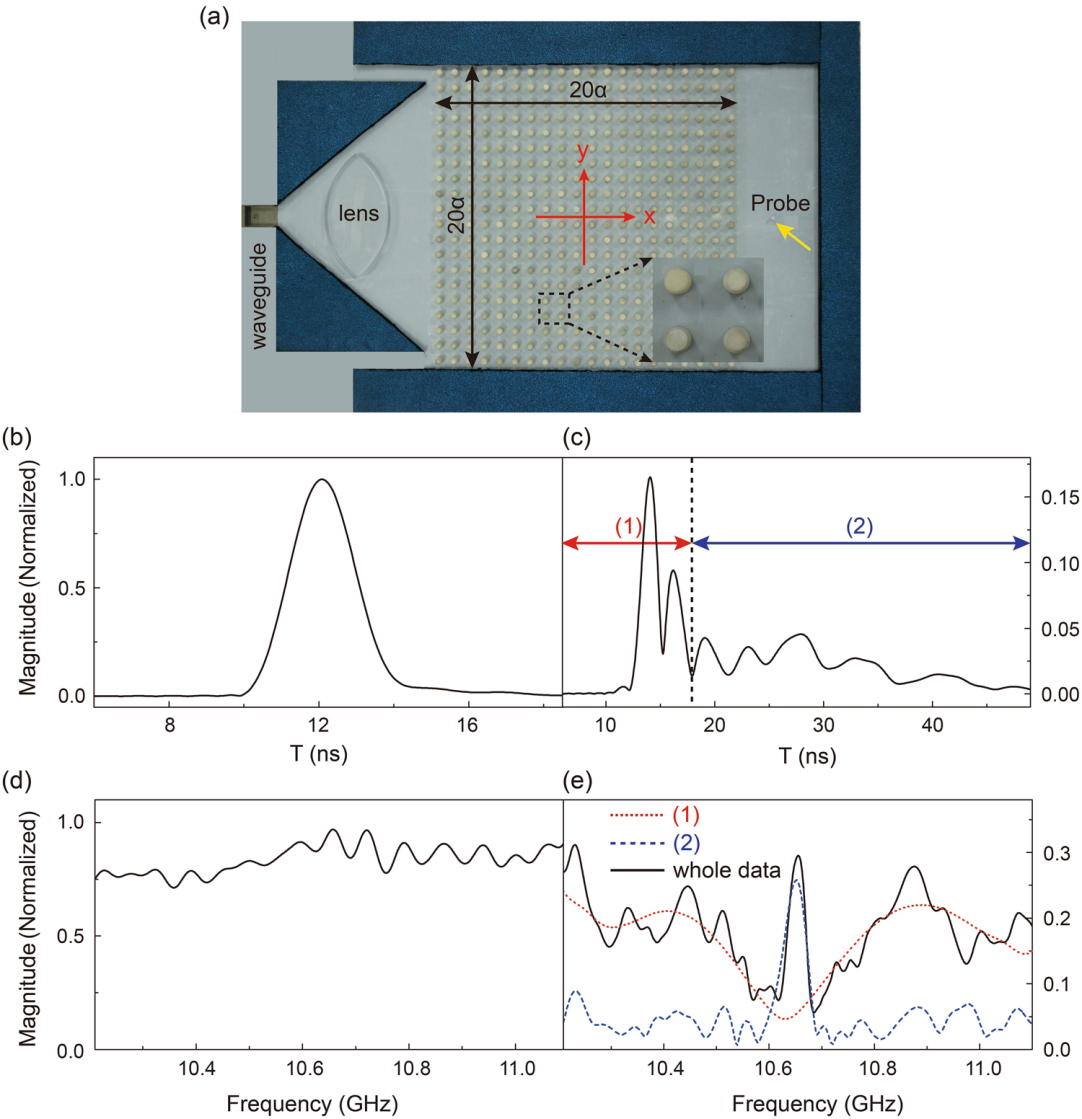
(**a**) Photo of our experimental setup. (**b**,**c**) The received pulse passing through the setup without/with the PC. (**d**) Related spectrum of the pulse without passing through the PC. (**e**) The red dotted line represents the spectrum of pulse (1), the blue dashed line represents the spectrum of pulse (2), and the black solid line represents the spectrum of the whole data.

Effective DZI parameters have successfully explained considerable interesting properties existing in DZI PCs. As it fails to explain the pulse reshaping effect, we conduct microwave experiments on PC structures (as illustrated in Fig. 2(a), see Methods). Due to the limitation of our experimental setup, the width and thickness of the PC slab are both set as 20*a*. A microwave pulse generated by our Vector Network Analyzer (VNA, Agilent E5071C) is incident from the X-band rectangle waveguide in gray with electric field polarized parallel to all cylinders. We first obtain the initial pulse incidence without the PC slab. Figure 2(b,d) are the measured temporal profile and its spectrum respectively, normalized to the largest value experimentally measured in time/frequency regime. The full width half maximal of the initial pulse is 2.01 ns. The pulse has a dramatic shape change after propagating through our PC slab (Fig. 2(c)). It is quite clearly that the pulse is divided into three parts, similar to our simulation results (Fig. 1(d,e)). To explore the cause of the pulse reshaping, we analyze the pulse by parts: (1) contains the first two peaks with an approximate duration of 6.00 ns and (2) contains the remaining part. We apply pulse-to-spectrum transformation in VNA to pulse (1) and (2), basically a short-time fast Fourier transformation (FFT) operation, and obtain the corresponding spectral distributions. As shown in Fig. 2(e), the spectra of (1) and (2) are quite different, neither bearing any similarity to that of the initial pulse, as shown in Fig. 2(d), without propagating through the PC slab. Though a not very precise spectrum is obtained due to the limited time duration for pulse (1), an obvious characteristic can be found that the center frequency part close to Dirac frequency *ω*_*D*_ is filtered. In contrast, only the frequency components close to *ω*_*D*_ appear in that of pulse (2), indicating that these frequencies, and only these frequency components leave the PC slab in an obvious later time. Even considering the whole duration of the experiment, including both pulse (1) and (2), its spectrum, as shown in the solid line in Fig. 2(e), does not recover its initial form. Certain frequency components cannot pass through the PC. For PCs with Dirac cones at the BZ boundaries, as the density of states at the Dirac point is limited, transmission through a finite-thickness sample is limited as well^1,10^, which means that the filtering effect as in pulse (1) is totally understandable. However, what is different here, from our pulse transmission experiment, is that the frequency components close to Dirac frequency *ω*_*D*_ indeed transmit through our DZI PC at a later time, whose frequency components are also consistent with those of the extra flat band intersecting the Dirac cone at the Dirac point.

**Figure 3:**
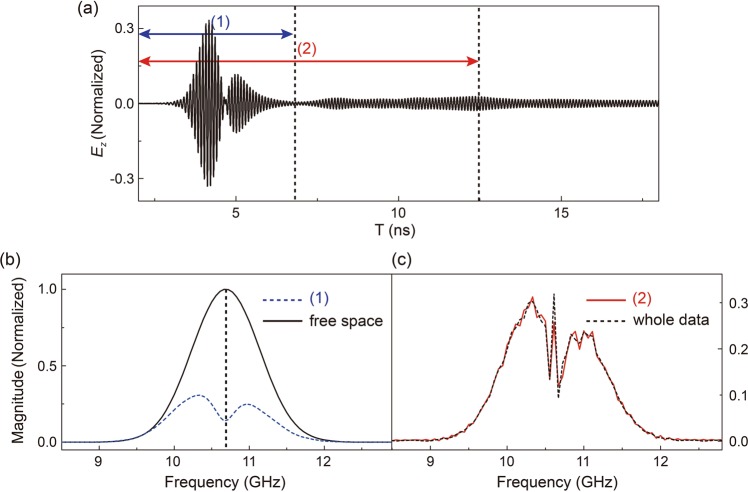
(**a**) Simulated temporal transmission spectra of the pulse after passing through the PC. (**b**) The black solid line represents the spectrum of the pulse propagating in the free space, and the blue dash line represents the spectrum of the first two peak of the pulse. (**c**) The red solid line represents the spectrum of the part (2), and the black dash line represents the spectrum of the whole data.

In order to provide a better understanding on our experimental results, simulations are also carried out based on the experimental situations. In simulations, a Gaussian-shape pulse, both in spatial and temporal domain, are considered as $\cos ({\omega }_{D})\exp [-{(t-{t}_{0})}^{2}/\Delta {t}^{2}]\exp [-{y}^{2}/{{w}_{0}}^{2}]$, where $\Delta t=0.5\,{\rm{ns}}$ is the temporal half-width of the pulse, ${w}_{0}=2{\lambda }_{0}$ is the spatial half-width, and ${\lambda }_{0}$ corresponds to the wavelength at the Dirac frequency ${\omega }_{D}$. The original temporal profile of the incident pulse shall be of a Gaussian shape [Fig. 3(b), solid line]. The width and thickness of the PC are set as 40*a* and 20*a*, respectively. We can observe that the pulse profile is reshaped after passing through the PC, as shown in Fig. 3(a), which is similar to the experimental result. By applying the FFT on the temporal profile, we can obtain the corresponding spectra shown in Fig. 3(b,c). The blue dash line represents the spectrum of the first two peaks of the pulse. Even though the initial incident pulse is of a different profile as in the experiment, by comparing with the spectrum of the incident pulse, a dip near the Dirac frequency is observed, indicating that the same filtering effect to frequencies close to *ω*_*D*_ occurs, which is quite similar to Dirac physics. In PC with Dirac cones at the BZ boundaries, the electromagnetic mode at the Dirac frequency cannot be excited due to the low density of states while a filtered pulse for the other modes in the Dirac cone shall be formed to propagate through, in the form of a Zitterbewegung-like phenomenon. As much more time is considered in the new pulse (2), similar as in experiment, the frequency components close to *ω*_*D*_ appear again, indicating that they reach the probe point at a much later time. Even with a much-increased length of the record time, owing to the higher precision in simulations than in experiments, the portion of these frequency components even increases, as shown in the dashed line in Fig. 3(c), while the frequency components corresponding to the Dirac cone remain unchanged. These modes are with a linear dispersion and shall arrive together with the same group velocity, equal to the slope of the linear Dirac cone dispersion. However, the modes belonging to the curly flat band [as in Fig. 1(a)], with frequencies very close to *ω*_*D*_, can have different group velocities and certain modes can arrive at even later time. It is now quite clear that the emergence of the third peak, the major difference of pulse reshaping phenomenon between Dirac cone and Dirac-like cone in PCs, is due to the flat band. However, very similar physics applies to either Dirac cone or Dirac-like cone for the first two peaks. As these modes correspond to the linear dispersion of the Dirac cone, a filtered pulse will be formed to propagate at a constant group velocity.

**Figure 4:**
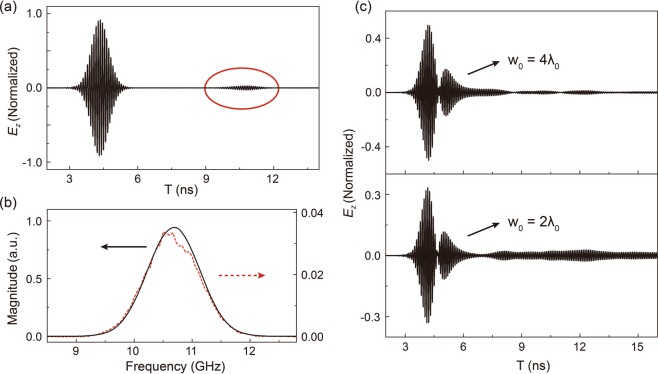
(**a**) Simulated temporal transmission spectra with plane wavefront. The second pulse due to multiple reflection in PC is encircled in red. (**b**) The spectra of the first (solid line) and the second pulse (dashed line). (**c**) The profiles of transmitted pulse through the sample with different transverse spatial half-widths.

To explore the excitation of the third peak, we consider different pulse incidence in simulations. Firstly, a temporal Gaussian packet is impinged into the PC, whose wavefront is planewave, not with Gaussian spatial distributions. Figure 4(a) plots the normalized profile of the output pulse. Interestingly, the shape of the pulse remains its temporal Gaussian profile (though its magnitude is slightly smaller than 1 due to the impedance mismatch between DZI PC to air). We observe an extra pulse signal (enclosed by a red eclipse) arriving after the first peak. By doing FFT on both signals, we may conclude that they both reflect the incident Gaussian frequency components, as can be seen in Fig. 4(b). The time difference between these two pulse signals is also dependent on the thickness of the PC sample. The emergence of the later signal shall be due to the multiple reflection of the incident pulse inside the DZI PC. As for the impedance mismatch between DZI PC and air, it eventually exits the PC slab and reaches the probing point. This will be elaborated later. Normal incidence with plane wavefront cannot reproduce the pulse reshaping in both experiments and simulations.

We further consider incident beams with different spatial distributions, namely different transverse spatial half-width *w*_0_. We can easily recognize in Fig. 4(c) that though the first two peaks, corresponding to the Dirac cone only, mainly remain their profiles. However, the third peak, corresponding to the flat band, is quite different. When we set *w*_0_ to $4{\lambda }_{0}$, the third part of the reshaped pulse emerges. However, if we decrease *w*_0_ to $2{\lambda }_{0}$, with much more oblique wave vectors, a much stronger excitation occurs, which can be easily understood from the shape of the flat band: with more incident transverse wave vectors, the flat band can have a better excitation. For normal incidence, only the linear dispersion in Dirac-like cone is excited without the excitation of the flat band and the original pulse profile maintains. On the other hand, when a spatial Gaussian pulse with oblique incident components is incident to the DZI PC, the oblique components will reflect from Dirac-like cone PC because of its close-to-zero index property and Snell’s Law. Away from Dirac frequency, the magnitude of effective refractive index increases and at the same time increases the portion of oblique components to refract into Dirac-like cone PC. This explains the filtering effect existing in the first two peaks that transmission extrema occurs to frequencies close to Dirac frequency. In this sense, up to here, the pulse reshaping phenomenon is similar to that of the Dirac cone at the BZ boundaries. This can only be understood in time domain. However, this is not the end of the story. Oblique incidence can also excite the extra flat band. These modes, though with much smaller and different group velocities, transmit and are received after propagating through the PC slab. This is also consistent with the fact that the third peak never appears in simulations with effective zero index parameters. Corresponding to the longitudinal electromagnetic excitation, this purely flat band for effective parameters can never be excited. Only after been discretized using PC structures, it will have slow but finite group velocities and appears later in time domain measurements.

### Group velocity inside a DZI PC

**Figure 5:**
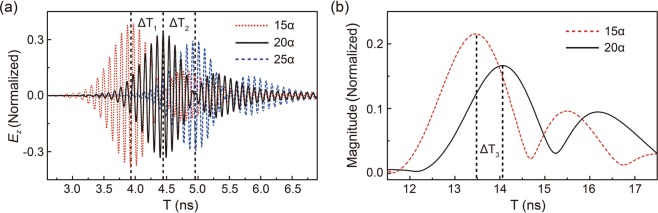
(**a**) Simulated temporal signals with Gaussian pulse incidence through DZI PCs with different thicknesses. (**b**) Experimental measured temporal signals through DZI PCs with different thicknesses.

Now we have a much better understanding of the mechanism behind the pulse reshaping through DZI PC. We can use the first two peaks of the pulse, corresponding to the excitation of the Dirac cone, namely the Dirac-like cone without the flat band, to measure the group velocity inside a DZI PC, because this part is with a linear dispersion and propagates with a constant group velocity. Zero-index is peculiar because of its infinitely large phase velocity, while the group velocity in ZIM has never been experimentally measured. In Fig. 5, we plot the profiles of the transmitted pulses after passing through PCs with different thicknesses. We set *w*_0_ as $2{\lambda }_{0}$ in simulations. The positions of both the incident port and the probe point are fixed as *L* in both experiments and simulations while the thickness of the PC between them is changed. For instance, the total time to propagate through a PC slab of thickness 15*a* is $15a/{v}_{g}+(L-15a)/c$, where *v*_*g*_ is the group velocity of DZI PC, *a* is the lattice constant and *c* is the speed of light in air. If we increase the PC slab thickness by 5*a* to 20*a*, the total time changes to $20a/{v}_{g}+(L-20a)/c$, and thus their time difference $\Delta {\rm{T}}=5a/{v}_{g}-5a/c$. The values of the delay time when PC slab thickness is increased by 5*a* are shown in Table 1, where we can easily obtain the group velocity of DZI PC as ${v}_{g}=5a/(\Delta {\rm{T}}+5a/c)\approx 0.349c$. It is well consistent with the slope of the calculated linear photonic dispersion (0.347*c*) near the Dirac-like cone, as shown in Fig. 1(b). Consistent result can also be found experimentally (0.340*c*).

**Table 1:** Delay time through PCs with different thicknesses.

ΔT_1_	0.525 ns
ΔT_2_	0.511 ns
ΔT = (ΔT_1+_ΔT_1_)/2	0.518 ns
ΔT_3_	0.539 ns

Knowing group velocity, we can then prove that the second pulse in Fig. 4(a) (encircled in red) is indeed from the multiple reflection between PC and air interfaces. The pulse interval ${\Delta {\rm{T}}}_{ref}$ between the main pulse and the second small pulse is 6.405 ns. Multiplying the pulse interval by the obtained group velocity, we obtain ${\Delta {\rm{T}}}_{ref}\times {v}_{g}\approx 40a$, which is double the thickness of the PC slab sample we used in simulations.

## Conclusions

In conclusion, we explore the pulse reshaping phenomena through DZI PC slabs with Dirac-like-cone dispersion. Through numerical simulations and microwave experiments, we find that peculiar pulse splitting occurs through a DZI PC slab with oblique incidence at frequencies close to Dirac frequency. A filtered pulse with constant group velocity appears first, where similar physics related to the Dirac cone at the BZ boundaries can be found. Another part of the pulse, corresponding to the extra flat band in the Dirac-like-cone dispersion can also be excited and distinguished. The group velocity close to Dirac frequency in DZI PC is experimentally measured where good consistence can be found with both numerical simulations and band diagram calculations. Besides the difference as an effective pseudospin-1/2 or a pseudospin-1 systems^35^, we believe that a new angle of view is thus provided to understand the difference between Dirac cone and Dirac-like cone in classical wave systems.

## Methods

### Numerical simulations

Throughout this paper, all numerical simulations are performed by COMSOL Multiphysics, 5.4, commercial software based on finite element method (www.comsol.com).

### Experimental setup

A photo of the experimental setup can be found in Fig. 2(a). A parallel-plate waveguide setup (the top metallic plate is not shown here) is used to guarantee a TM polarization, with electric fields polarized along the *z* axis. Our photonic crystal is composed of a square array of alumina cylinders with radius R = 3.75 mm, height = 11 mm (also the distance between parallel metallic plates to clad our sample) and the dielectric constant ε = 8.2. The lattice constant *a* = 16.63 mm and all parameters reflect our numerical simulations considered in Fig. 1. Accidental triple-degeneracy with a Dirac-like cone whose Dirac frequency ${\omega }_{D}=10.682\,{\rm{GHz}}$ can thus be achieved, as shown in Fig. 1(c). Absorbing materials (in blue) are used to surround the PC structure to avoid unwanted scattering. Using a home-made acrylic lens, the microwave impinging from an X-band rectangle waveguide port (in grey) transforms into a Gaussian beam with a beam width of approximately 80 mm. A dipole antenna indicated by a yellow arrow is placed outside the sample to measure the transmitted signal. Different from our previous microwave experiments where time-harmonic field distributions were measured, as it is very difficult to obtain the real time distribution of pulse reshaping inside the DZI PC as illustrated in Fig. 1(d), we customize our VNA to emit a pulse with center frequency at ${\omega }_{D}=10.682\,{\rm{GHz}}$ and collect the temporal signal at a single point as in Fig. 1(e) instead.

## Data Availability

The data in this study are available from the authors upon reasonable request.
